# Heavy metal removal with magnetic coffee grain

**DOI:** 10.3906/kim-2006-47

**Published:** 2021-02-17

**Authors:** Sevgi ASLIYÜCE ÇOBAN, Ivo SAFARIK, Adil DENİZLİ

**Affiliations:** 1 Hacettepe University, Department of Chemistry, Ankara Turkey; 2 Department of Nanobiotechnology, Biology Centre CAS, Ceske Budejovice Czech Republic; 3 Regional Centre of Advanced Technologies and Materials, Palacky University, Olomouc Czech Republic

**Keywords:** Magnetic modification, coffee grain, heavy metal ion, synthetic waste water

## Abstract

The presence of heavy metals in environmental waters having an important place in the industrial waste is a major threat to viability. Heavy metals are transported to humans through the ecological cycle, damaging many tissues and organs. In recent years, agricultural and food waste can be used to remove heavy metals. At the present study, magnetically modified coffee grains which are alternative to conventional particle systems were prepared and heavy metal removal performances were investigated. The coffee grains used were magnetically modified by contact with water-based magnetic fluid. Magnetically modified coffee grains were characterized by scanning electron microscopy (SEM), Brunauer–Emmett–Teller (BET) surface area analysis and electron spin resonance (ESR). Adsorption studies are made with four different heavy metal ions, namely Cu(II), Pb(II), Cd(II) and Zn(II). Adsorption isotherms were determined and heavy metal removal performance of magnetic coffee grains were investigated from synthetic waste water.

## 1. Introduction

In recent years heavy metal pollution has reached dangerous dimensions with the increase in industrialization. Many industrial activities aiming at improving the quality of life of people also leave heavy metals to the environment, which will endanger for human health. Fertilizers, pesticides, mining, tanning and electronics are industrial branches that produce large amounts of heavy metals [1,2]. In addition to the rapidly growing electronics market, the increasing amount of electronic waste has increased the heavy metal pollution. The major polluting heavy metals are cadmium (Cd), chromium (Cr), mercury (Hg), lead (Pb), copper (Cu), aluminum (Al), nickel (Ni) and zinc (Zn) [3–5]. These metals, which are transmitted to the environmental waters, accumulate in plant tissues and insert animal feed in the food chain. Heavy metal contaminated plants are also indirectly involved in animal foods by entering the food chain of the animals and finally reaching the human being found at the top of the food chain [6]. Heavy metals, which are generally not biodegradable, accumulate in many tissues and organs such as lung, kidney, liver, pancreas, nerve and cause many diseases [7,8].

Coagulation, reverse osmosis, membrane filtration, ion exchange, photocatalysis and electrochemical applications are some of the traditional methods commonly used to remove heavy metals from waste water [9,10]. Although these methods are capable of effective separation, they have considerable disadvantages due to their high labor and cost as well as their secondary waste generation. According to the recent studies, various adsorption techniques are promising compared to traditional methods. Direct or modified use of bacteria, fungi, algae and plants as adsorbents of biological origin has been the focus of interest in recent years [11–13]. Since all of the biological structures contain many functional groups such as amino, hydroxyl, phosphate groups, they can have binding and carrier properties.

The use of food and agricultural wastes as biological adsorbents is a new environmentally friendly approach. Coffee can be used as one of these adsorbents, which is consumed in millions of tons per year all over the world. Although some of these coffee wastes are reused as animal feed, most coffee wastes are burned for disposal and generate greenhouse gas. There have been several methods developed for the reuse of coffee grains in recent years, such as D-mannitol raw materials for the food industry, biodiesel and bio-sorbent [14]. Previously, coffee grains have been used as a biosorbent in dye and metal treatment [15,16]. In contrast to the present study, magnetic modification was not used in metal removal studies with coffee grains.

The advantages of biologically based wastes can be increased by changing their properties with various modifications. One of these modifications is the addition of magnetism to the structure. Magnetic separation is a promising technique for adsorption of target compounds from complex samples (such as blood, plasma, growth media, wastewater). Magnetic nano- and microparticles have been applied in various fields of biotechnology, analytical chemistry and environmental technology [17,18]. Magnetic materials have been shown to be successfully used in many studies for water treatment [19,20]. The magnetic particles combine the advantages of fluidized beds and filled columns. These materials provide low pressure drop and high feed current, such as fluidized beds, while high mass transfer rates and high liquid-solid contact, such as filled columns. In this way these materials are easily removed from the media with magnetic separators. Studies have been carried out for various magnetic modified biological wastes such as corn straw, sawdust, sugar cane bags and waste tea leaves as well as pomelo, orange, litchi shells and eggshell powder for heavy metal adsorption [21–24].

In this study, we present magnetic modification of spent coffee grains to develop environmentally friendly heavy metal adsorbents without creating secondary waste.

## 2. Materials and methods

### 2.1. Materials

Coffee grains were obtained from local coffee establishments in Czechia. Nitrated compounds of heavy metals used in adsorption studies were purchased from Merck KGaA (Darmstadt, Germany). Ethylene diamine tetra acetic acid (EDTA) and HNO_3_were purchased from Sigma-Aldrich Corp. (St. Louis, MO, USA). Distilled water was used for all prepared solutions.

### 2.2. Magnetic modification of coffee grains

Perchloric acid stabilized water-based magnetic fluid was prepared as described in the literature [25]. Ferrofluid consists of magnetic iron oxide nanoparticles with diameters ranging from 10 to 20 nm (electron microscope measurements). The relative magnetic fluid concentration (about 25 mg L^-1^) is given as iron oxide content determined by a colorimetric method [26]. Magnetic modification was performed as described recently [16]. Ten g of spent coffee grounds was first added into 80 mL of methanol. Ten mL of magnetic fluid was then added to this suspension and stirred. After stirring for 1 h, the coffee grains were washed with methanol and dried at room temperature. Dried coffee grains are ready for characterization.

### 2.3. Characterization of magnetic coffee grains

The SEM images of the modified and dried coffee grains were examined for their pore structure. The dried coffee grains were coated with gold film and then scanned by SEM (JSM 5600, JEOL Ltd., Tokyo, Japan) at various magnifications. Electron spin resonance spectrometer (EL 9, Varian Medical Systems Inc., Palo Alto, CA, USA) was used to determine the magnetic properties of coffee grains. Finally, the coffee grains in the aqueous solution were optically photographed in the presence and absence of a magnet.

### 2.4. Heavy metal adsorption from aqueous solutions

Four different metals, Cd(II), Pb(II), Cu(II), Zn(II), were studied to investigate the adsorption of heavy metal from the aqueous solution of magnetic coffee grains. All experiments were performed in the batch system at 100 rpm in a rotator for 2 h with 10 mg of magnetic coffee grains and 20 mL of ion solution. The solutions were prepared with nitrate salts of selected heavy metals in the range of 6–200 mg/L. To determine optimum adsorption conditions, experiments were performed for each metal ions at different pH (2–8) ranges. Each experiment was repeated three times. The metal ion concentration in the samples which were not treated and for 2 h treated with coffee grains were measured by flame atomic adsorption spectrometry (AAS) (Shimadzu AA-6800 Flame, Shimadzu Corp., Kyoto, Japan) including deuterium background correction. Lead, cadmium, zinc and copper hollow-cathode lamps operating at 283.3 nm, 213.9 nm, 248.3 nm, 324.8 nm were used as light sources and the lamp currents were 10 mA, 4 mA, 15 mA, 15 mA, respectively. The spectral slit widths were 0.2 nm. All samples were measured in triplicate and the AAS was standardized with known ions of concentration at certain intervals. The amount of adsorbed metal ions was calculated by Equation 1. According to the equation; M refers to the amount of heavy metal in the initial and final solutions, and m refers to the weight of dry coffee grains.

(1)Q=(Minitial-Mfinal)m

### 2.5. Desorption studies

Two different desorption solutions were tested to remove the heavy metal ions adsorbed to the magnetic coffee grains. Desorption rates were determined using 0.1 M HNO_3_and 0.1 M EDTA for 2 h separately after heavy metal ion adsorption studies. Then, 10 consecutive repeated adsorption desorption experiments were performed to determine the desorption solution causing the least decrease in adsorption capacity and the selected appropriate desorption solution was used in all adsorption experiments.

### 2.6. Heavy metal adsorption from synthetic waste water

First, a 10 mL aqueous solution was prepared containing 50 mg/L of Ni (II), Fe (III), Co (II), Sn (II), Ag (II), Co (II). Each of the metal ions used in the adsorption studies [Cd (II), Zn(II), Cu (II) and Pb (II)] were added to this solution at a final concentration of 0.5 mmol/L. Finally, NaCl was added to the solution such that the amount of solution was 700 mg/L. Heavy metal adsorption from synthetic wastewater was carried out in a batch system. The amount of metal ion adsorption of the magnetically modified coffee grains from the competitive environment was calculated as specified in Section 2.4.

## 3. Results and discussions

### 3.1. Characterization of magnetic coffee grains

**Figure 1:**
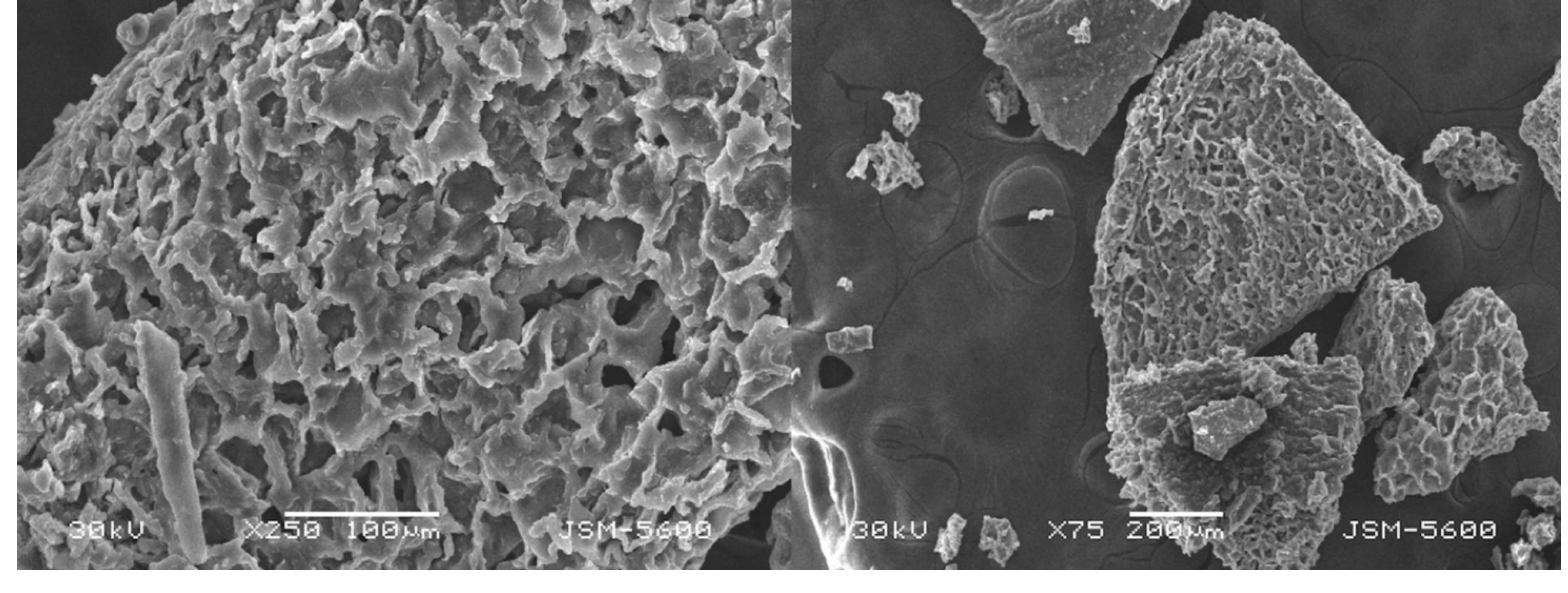
SEM images of magnetic coffee grains.

When SEM images were examined (Figure 1), it was observed that magnetically modified coffee grains had pores with a diameter of 10–30 µm. This natural porous structure facilitates mass transfer which is of great importance for adsorption. Furthermore, its porous structure provides a large surface area for adsorption. According to the BET results, the surface area of the magnetic coffee grains was found to be 4.98 m^2^/g. Coffee grains have similar surface areas when compared to materials used in adsorption experiments [13]. According to these results, magnetic coffee grains have sufficient surface area for adsorption.

**Figure 2:**
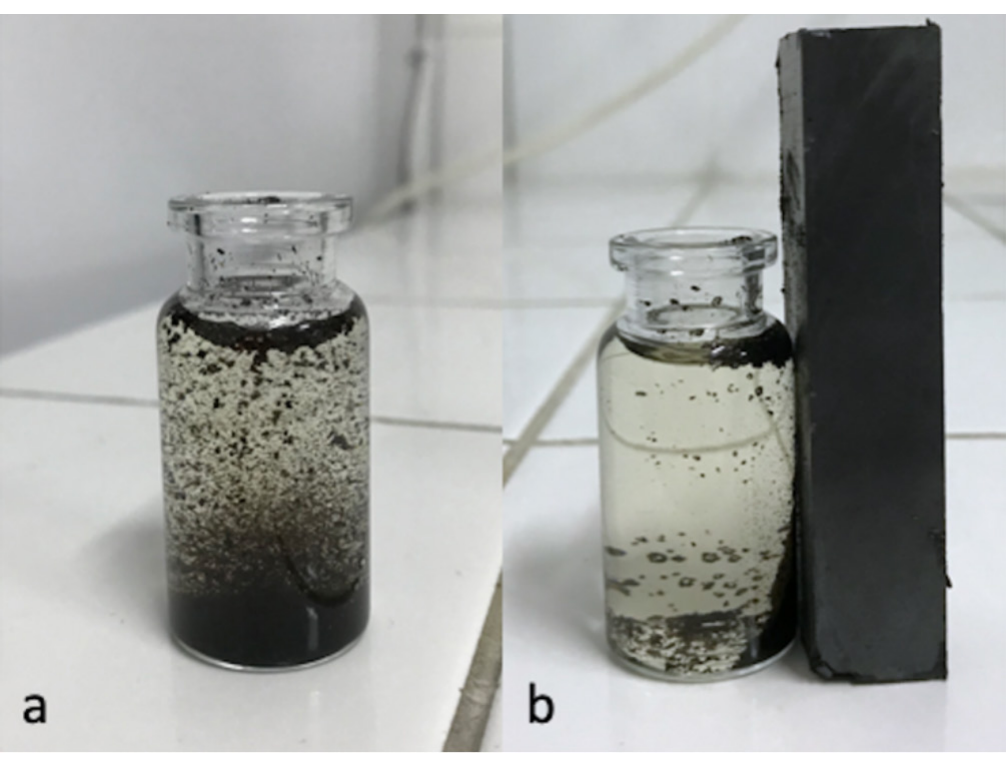
(a) Optic images of magnetic coffee grains; (b) optic images of magnetic coffee grains under the influence of magnet.

When the optical images of the magnetic coffee grains are examined, it is seen that, while the coffee grains that do not interact with the magnet are suspended in aqueous solution (Figure 2a), when they interact with the magnet they are collected in this direction (Figure 2b).

**Figure 3:**
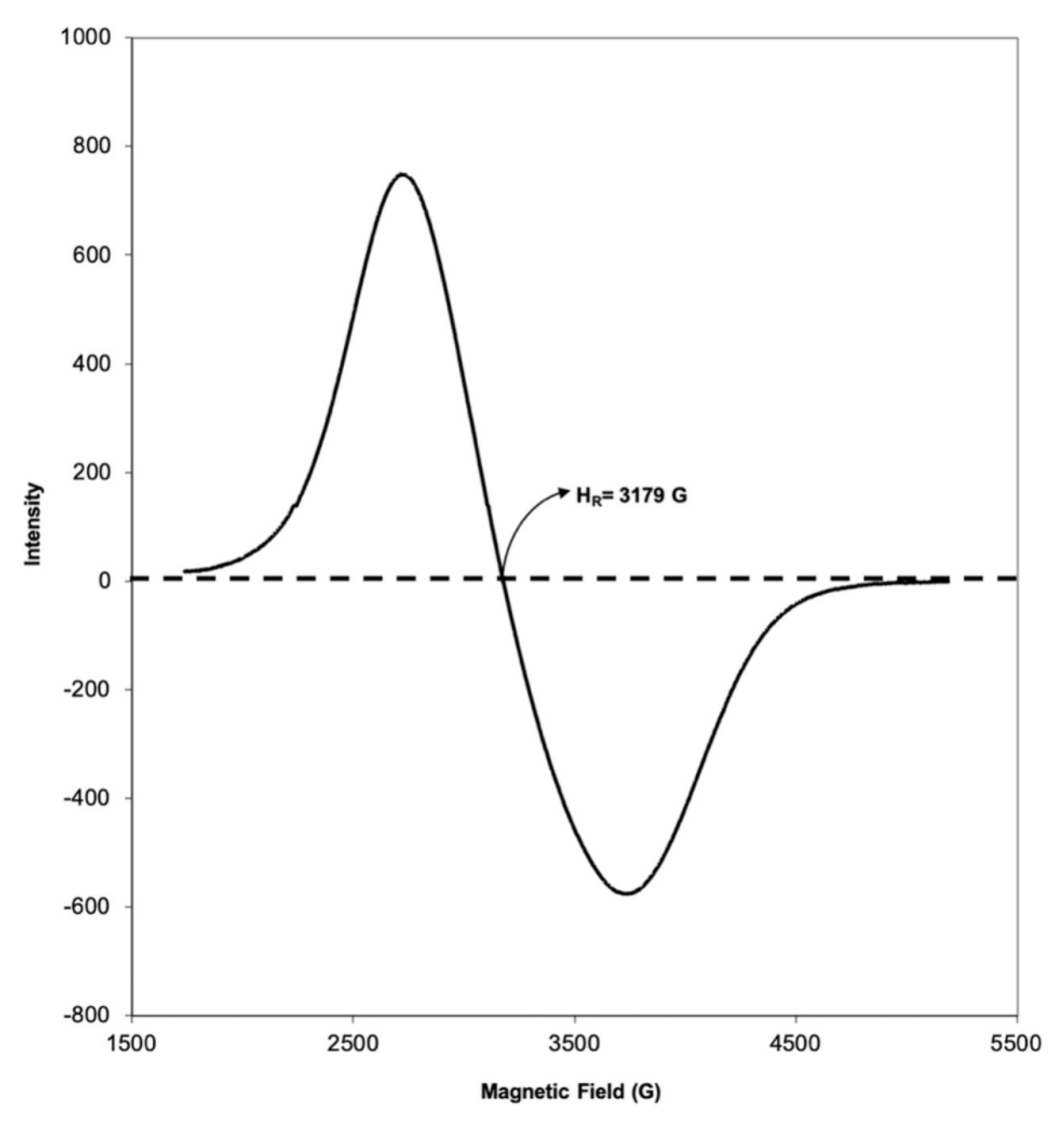
ESR spectrum of magnetic coffee grains.

The ESR spectrum shows the magnetic field behavior caused by the magnetization of the materials. Accordingly, when the ESR spectrum was examined (Figure 3), it was observed that the external magnetic field applied to the magnetically modified coffee grains increased with the local magnetic field. This magnetic field strength decreases over time and reaches zero. The value at the point where it reaches zero is called the resonance magnetic field (*Hr*). This value refers to the magnetic field required per mass of the sample to stimulate the magnetic materials in the sample.*Hr*value for magnetic coffee grains was found to be 3179 Gauss.

Another value for explaining the magnetism of the materials is*g*. This value can be explained as the quantity property of molecules with unpaired electrons and calculated according to the previous study [27]. From the calculations, the*g*value of magnetic coffee grains was found to be 2.2, which is in the range of the values of magnetic materials in the literature. Compared with the literature, Fe (III) values for low and high spin complexes were reported to be in the range of 1.4–3.1 and 2.0–9.7, respectively [28].

### 3.2. Heavy metal adsorption from aqueous solutions

**Figure 4:**
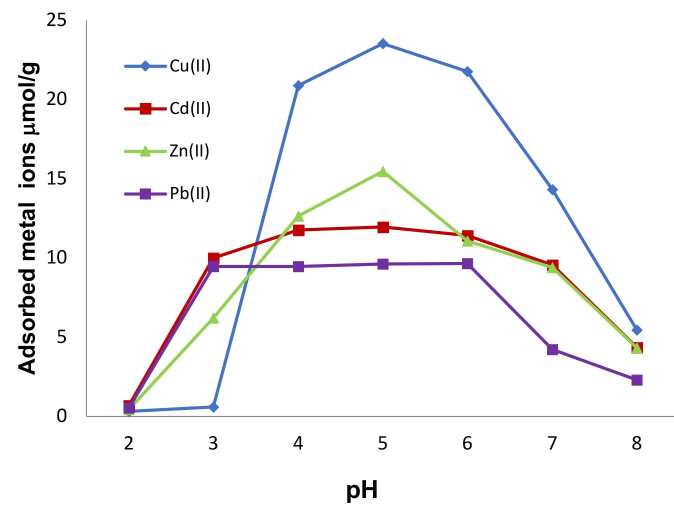
Effect of pH on heavy metal adsorption; heavy metals concentration: 10 mg/L; time: 120 min; T: 25 °C.

The effect of pH on the adsorption of metal ions results from the behavior of metal ions in aqueous solution. Adsorption shows a significant increase for all metal ions from pH 2 to pH 3 (Figure 4). The complete dissolution of metal ions significantly affects the amount of adsorption. Therefore, the highest adsorption was observed in the range of pH 4–6 for Cu(II) and Zn(II). The highest values found for these metals are about 23.5 and 15.5 µmol/g respectively. On the other hand, Cd(II) and Pb(II) differs from these metal ions and shows effective adsorption in the pH range 3–6 and the highest adsorption was found to be 9.5 and 12 µmol/g.

**Figure 5:**
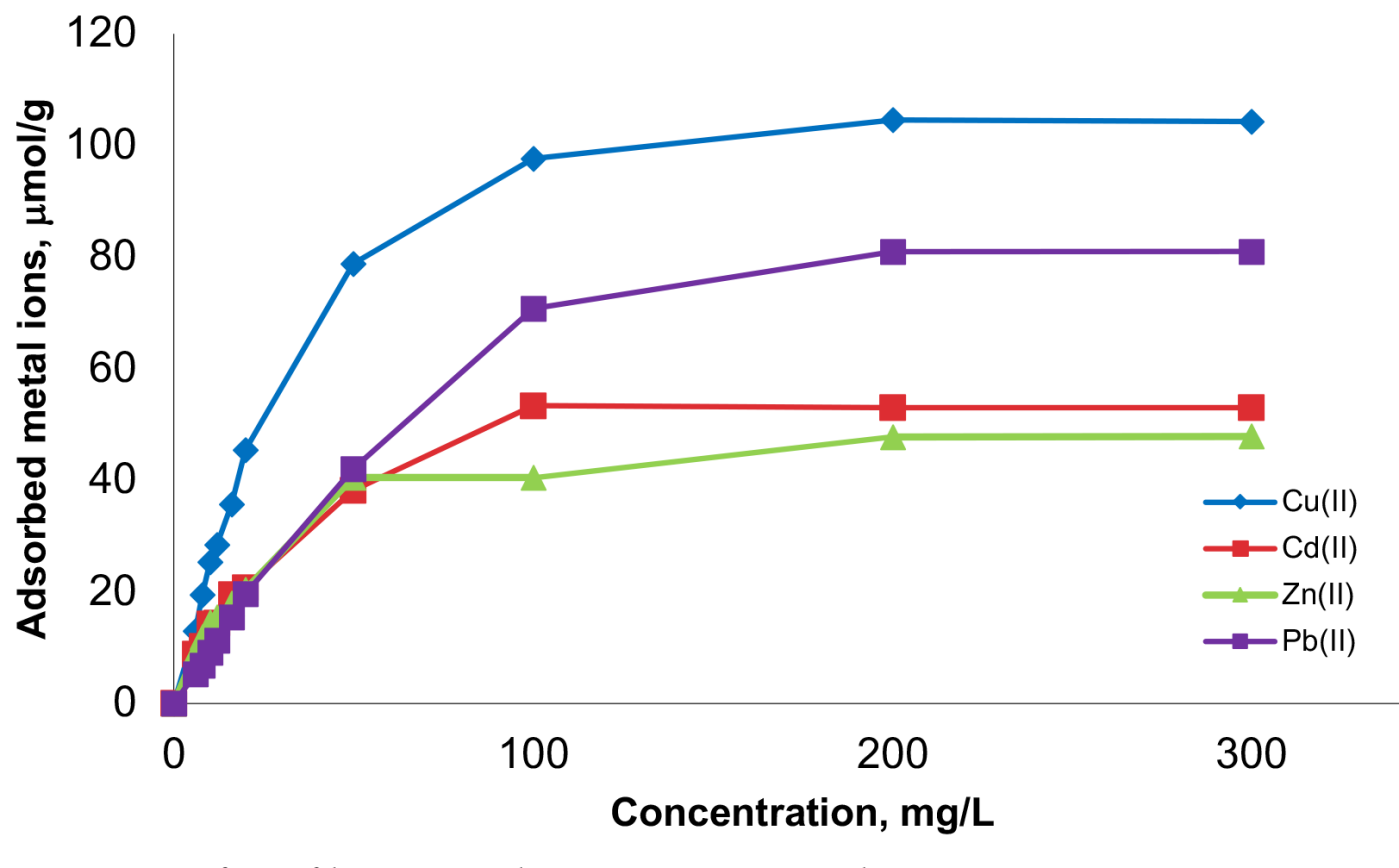
Effect of heavy metal concentration on adsorption capacity; pH: 5.0; time: 120 min; T: 25 °C.

Experiments with 9 different initial concentrations to determine the adsorption capacity of magnetic coffee grains; adsorption was carried out at pH 5, which was the most frequent pH. In individual experiments with initial concentrations of 6, 8, 10, 12, 16, 20, 50, 100, 200, and 300 mg/mL for four metal ions at room temperature, adsorption for each metal ion decreased after 20 mg/mL. It was observed that 100 mg/mL slowed down well and stabilized at 200 mg/mL. This means that the binding regions on the surface of the magnetic coffee grains are filled. Among the metal ions, the highest adsorption value in micromol was observed for Cu(II). This is followed by Pb(II), Cd(II) and Zn(II). The highest adsorption values of metal ions to magnetic coffee grains are about 104, 81, 53, 48 µmol/g, respectively (Figure 5). Table 1 indicates the comparison of heavy metal adsorption capacity of the different coffee-based sorbents used in literature [29–33].

**Table 1:** The comparison of heavy metal adsorption capacity of the different coffee-based sorbents used in literature.

Adsorbent	Qmax(µmol/g)				Ref.
	Cu	Cd	Zn	Pb	
Coffee grounds	-	139.2	-	-	[29]
Degreased coffee beans	-	59.9	-	-	[30]
Coffee grounds	-	-	-	41.7	[31]
Coffee residues blended with clay	265.76	-	-	-	[32]
Coffee husks	116.5	61.38	84.2	-	[33]
Magnetically modified coffee grain	104	53	48	81	in this study

### 3.3. Adsorption isotherms

Adsorption is similar to an equilibrium reaction. When the solution is brought into contact with a certain amount of adsorbent, the concentration of the adsorbed material in the solution is reduced until it equilibrates with those on the adsorbent surface. After the adsorption equilibrium is established, the concentration of the adsorbed material in the solution phase remains constant. Generally, the amount of adsorbed material is determined as a function of concentration at constant temperature. At constant temperature, adsorption isotherm is obtained by crossing the amount of adsorbed solute in unit weight of adsorbent against the concentration of solute remaining in solution in equilibrium. In this study, Freundlich and Langmuir adsorption models were used to examine the adsorption isotherms. The equations used for these two isotherm models are given below respectively.

(2)lnQe=1n(lnCe)+lnKF

(3)1Qe=(1Qmax)+(1Qmaxb)(1Ce)

Eq. 2 represents the Freundlich isotherm model. According to this equation, Q_e_is theoretically the amount of analyte adsorbed per unit of stationary phase, C_e_is the analyte concentration in equilibrium, K_F_is the adsorption capacity and n is the adsorption intensity. According to the Freundlich model, the increase in the concentration of the analyte in the mobile phase increases the amount of material bound to the surface of the solid phase. According to the Freundlich model, it is assumed many regions on the adsorbent surface with different adsorption energies.

Eq. 3 is used to express the Langmuir adsorption model. In this equation, unlike Eq. 1, Q_max_represents the highest adsorption capacity of the fixed phase and b represents the tendency of the binding regions. The Langmuir isotherm assumes that the adsorbent surface has a constant number of active regions with the same energy and the adsorption energy is constant. In addition, adsorption occurs as a single layer, and the maximum adsorption is the adsorption at the time the molecules bound to the adsorbent surface form a saturated layer.

Langmuir and Freundlich isotherms values are given in Table 2. The R2 values calculated for both isotherms according to these values are closer to 1 in the Langmuir isotherm model although they are close to each other. On the other hand, Q_max_values for Cu(II), Cd(II), Zn(II), Pb(II) were obtained as 109.1µmol/g, 77.50 µmol/g, 49.77 µmol/g, 82.62 µmol/g respectively adsorption capacity was calculated as 104.5 µmol/g, 53.02 µmol/g, 47.77 µmol/g, 80.88 µmol/g and the theoretical Q_max_value expressed by the Langmuir isotherm is close to the maximum adsorption capacity found experimentally. In this case, it can be said that the adsorption event in the experiment is more appropriate to the Langmuir isotherm model.

**Table 2:** Langmuir and Freundlich isotherms for magnetic coffee grains.

	Experimental	Langmuir			Freundlich		
	Q(µmol/g)	Qmax(µmol /g)	b	R2	KF	1/n	R2
Cu (II)	109.1	104.5	0.1409	0.9956	1.326	0.3877	0.5586
Cd (II)	77.50	53.02	0.0606	0.8898	1.343	0.5848	0.9647
Zn (II)	49.77	47.77	0.0949	0.995	1.61	0.3333	0.9469
Pb (II)	82.62	80.88	0.168	0.9842	2.73	0.4033	0.9603

### 3.4. Adsorption dynamics

Adsorption kinetics were investigated to investigate the adsorption mechanism, to determine the adsorption rate and to calculate the maximum adsorption capacity of magnetic coffee grains for the adsorption of heavy metals. In this study, two well-known kinetic modeling pseudo-first order (Eq. 4) and pseudo-second order kinetic models (Eq. 5) were analyzed and used according to the equations given below.

(4)log(qe-qt)=logqe-k1t2.303

(5)(tqt)=(1k2qe2)+(1qe)t

In Eqs. 4 and 5, q_t_(µmol/g) and q_e_(µmol/g) respectively represent t adsorption capacity at contact time and equilibrium. k_1_and k_2_are first and second order rate constants. When log (q_e_-q_t_) values are plotted against t values according to equation 3, the velocity constant k_1_is calculated from the slope value of the equation obtained and the theoretical q_e_value is calculated from the cut-off point. When t/q_t_is passed against t values according to equation 4, the theoretical q_e_from the slope value and the speed constant k_2_from the cut-off point can be calculated. The kinetic data obtained as a result of this study are given in Table 3.

**Table 3:** Pseudo-first and second order kinetic values of magnetic coffee grains.

Heavy metal	Pseudo-first order			Pseudo-second order		
	Qeq(µmol/g)	k1(1/min)	R2	Qeq(µmol/g)	k2(g/ µmol.min)	R2
Cu (II)	17.5	0.061	0.9347	26.9	1.7088	0.9983
Cd (II)	13.7	0.055	0.9967	15.6	1.795	0.9968
Zn (II)	48.0	0.035	0.6299	15.3	1.005	0.9975
Pb (II)	6.46	0.058	0.9926	9.18	0.105	0.9996

Table 3 shows that the correlation coefficients of pseudo-first order and pseudo second order adsorption kinetics for Cu(II), Cd(II), Zn(II), Pb(II) are quite high. However, the q_e_values calculated for the pseudo-second order kinetic model seem to be quite close to the experimentally obtained q_e_values; the experimental q_e_values for 10 ppm initial for Cu(II), Cd(II), Zn(II), Pb(II) concentration were 25.3 µmol/g, 14.2 µmol/g, 14.5 µmol/g, 9.18 µmol/g, while the theoretical q_e_values were 26.9 µmol/g,15.6 µmol/g, 15.3 µmol/g, 9.60 µmol/g respectively. Thus, it can be said that the adsorption of metal ions on the surface of magnetic coffee grains is suitable for pseudo-second order kinetic model.

### 3.5. Heavy metal adsorption from synthetic waste water

In order to determine the metal retention behavior of the heavy metal removal activities of magnetic coffee grains in the multimetal containing medium, an artificial medium was prepared simulating industrial waste water. In multiple environments, the parameters increase affecting the adhesion of metal ions to the matrix surface. In addition to pH, temperature, metal concentration, adsorbent surface properties; it also depends on the combination of metals with each other and the number of metal ions competing for the binding sites. In the solution of such multiple metal ions, the adsorbent either does not react or shows two different reactions, namely the synergistic and antagonistic effect. Synergism means that the effect of the mixture is greater than the single effect of each metal ion and antagonism means that the effect of the mixture is less than the single effect of each metal ion.

**Figure 6:**
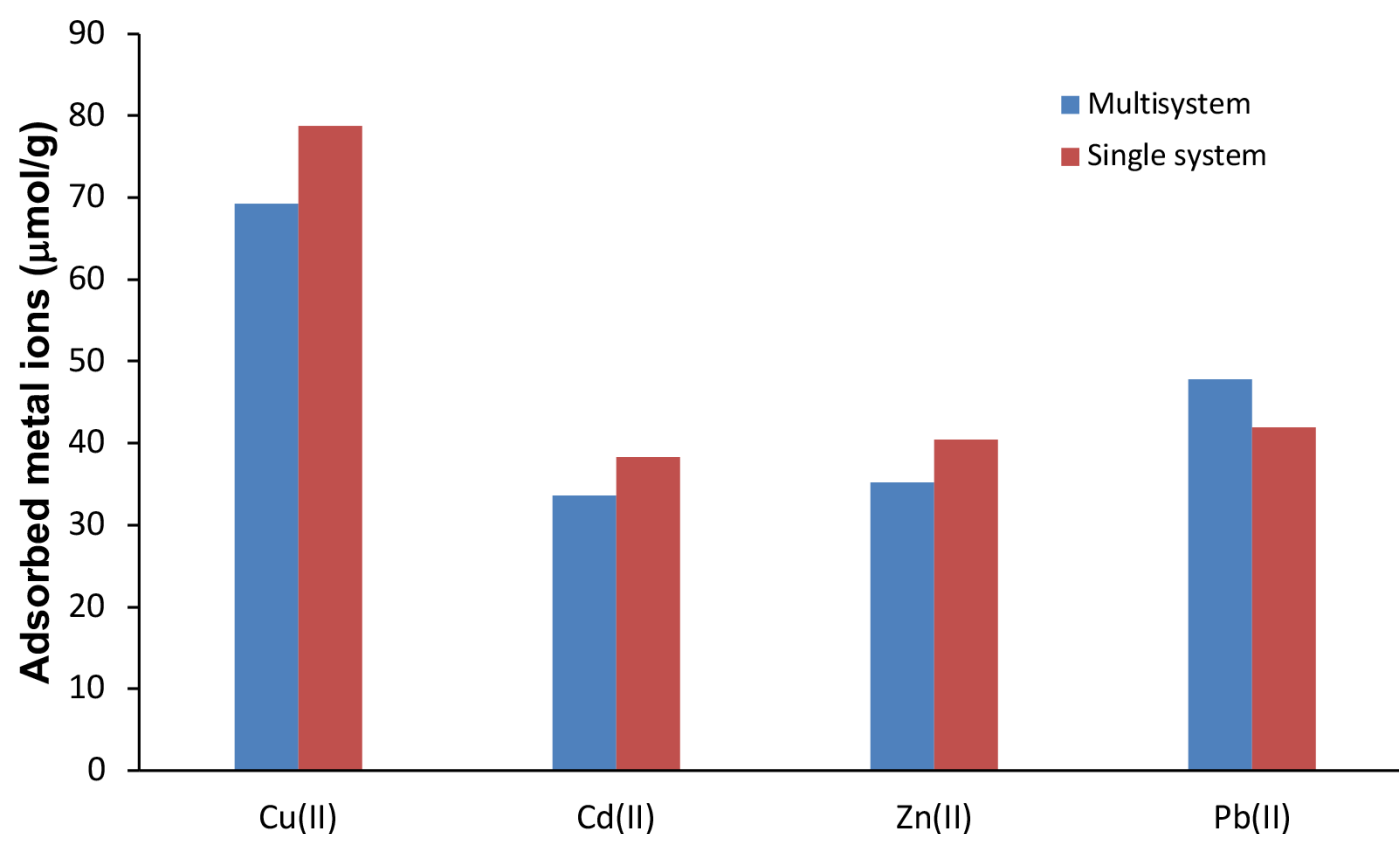
Competitive adsorption from synthetic wastewater.

The adsorption capacity for Cu(II), Cd(II), Zn(II) and Pb(II), respectively, in the single medium was about 78, 38, 40, 42 µmol/g, while in the multiple medium 69, 33, 35, 47 µmol/g was obtained. As shown in Figure 6, the adsorption of Cu(II), Cd(II) and Zn(II) to magnetic coffee grains decreased in comparison to the single medium. Unlike these three metal ions, the adsorption capacity of magnetic coffee grains to Pb(II) ions in multiple media increased synergistically compared to the single medium.

### 3.6. Desorption and reusability

**Figure 7:**
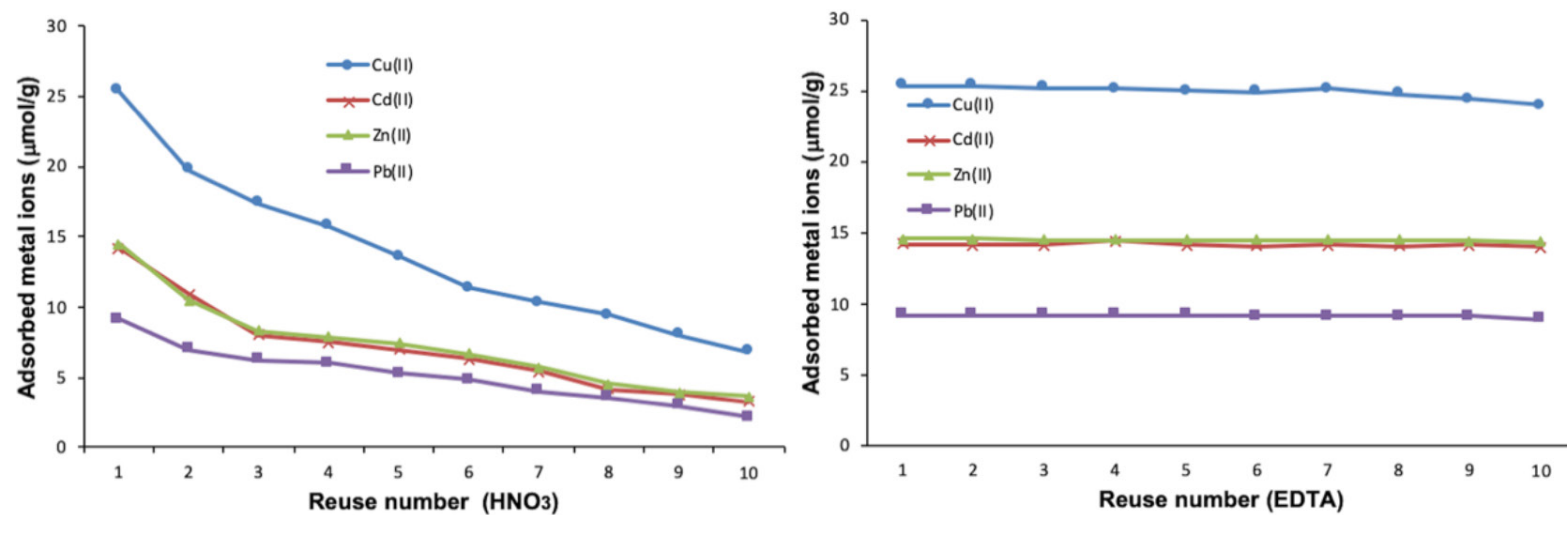
Reusability of magnetic coffee grains; (a) desorption solution: 0.1 M HNO_3_; (b) desorption solution: 0.1 M EDTA.

EDTA and HNO_3_which are the most commonly used to remove heavy metal in the literature were tried to remove the effect of metal ions adsorbed from the surface of magnetic coffee grains and to determine the appropriate desorption agent to ensure the long-term use of magnetic coffee grains. In the experiments where EDTA was used as the desorption agent after 10 times of adsorption desorption cycle, 5% decrease was observed in the adsorption capacity, and approximately 73% decrease was observed as a result of desorption with HNO_3_(Figure 7). According to the results, it was determined that HNO_3_is not suitable desorption agent for coffee grains which is a biological adsorbent. This is thought to be due to the fact that HNO_3_, a strong acid, damages biological functional groups that bind heavy metal ions to coffee grains. 0.1 M EDTA is a suitable desorption agent for effective long-term use of coffee grains in this experiment.

## 4. Conclusion

In this study, coffee grains, which are the food waste, are easily magnetized and the removal of heavy metals, which are the major risk factor for living, from wastewater is carried out on the laboratory scale. The ability of magnetic coffee grains to adsorb these metals in a multimedia environment was also found to be quite successful in experiments with synthetic waste water. As desorption agent, EDTA was chosen as the suitable agent for effective desorption and reusability of coffee grains. Thus, both the waste products were recycled and the efficient, environmentally friendly material for removing heavy metals were introduced.
